# Cultural Adaptation of the teen Mental Health First Aid (tMHFA) Program from Australia to the USA

**DOI:** 10.1007/s12310-023-09576-z

**Published:** 2023-04-17

**Authors:** Lacey L. Rosenbaum, Sanjana Bhakta, Holly C. Wilcox, Elise T. Pas, Karen Girgis, Aubrey DeVinney, Laura M. Hart, Sarah M. Murray

**Affiliations:** 1 Mental Health First Aid, National Council for Mental Wellbeing, Washington, DC USA; 2 Department of Mental Health, Johns Hopkins Bloomberg School of Public Health, Baltimore, MD USA; 3 Centre for Mental Health, Melbourne School of Population and Global Health, Melbourne, Australia; 4 School of Psychology and Public Health, Le Trobe University, Melbourne, Australia; 5 Mental Health and Resilience Group, Cheverly, MD USA; 6 International Psychology Department, The Chicago School of Professional Psychology, Washington, DC USA

**Keywords:** Adolescents, Secondary schools, Mental health education, Cultural adaptation, Contextual adaptation

## Abstract

teen Mental Health First Aid (tMHFA) is an evidence-based program developed in Australia that teaches young people in grades 10–12 how to identify and respond to signs of mental health challenges and crises among peers. Recognizing the growing adolescent mental health crisis in the USA, the National Council for Mental Wellbeing, in partnership with a Johns Hopkins University research team, used a multimethod research approach to adapt the program culturally and contextually from Australia to the USA. The goals of the study were to engage adolescents, MHFA instructors, and content area experts (*N* = 171) in a process to determine: how to retain the elements of the course that were evidence-based and effective while adapting the program for US students, what topics to add so US students have the essential information and skills teens needed to help a friend experiencing a mental health challenge or crisis, what changes to make to curriculum materials to ensure the style and delivery resonate with US students, and what tools to include so the program is implemented safely and with fidelity in diverse US schools. This paper outlines the adaptation process, including engaging participants, identifying key recommendations for modification, and making changes to the tMHFA program. The findings demonstrate the types of adaptations that may be needed to facilitate implementation and maintenance of program effectiveness when introducing tMHFA to new populations of students in the USA. In addition, the process outlined can be replicated toward this purpose as the program continues to expand both in the USA and in other countries.

## Introduction

Rates of depression, anxiety, self-harm, and suicide among adolescents in the USA were rising prior to the COVID-19 pandemic. Survey data from the National Survey on Drug Use and Health (NSDUH) show that teens ages 12–17 experiencing a Major Depressive Episode (M.D.E.) increased from 9.0 percent in 2004 to 15.7 percent (or 3.8 million people) in 2019 (SAMHSA, 2020). This trend is consistent with dramatic increases in suicide attempts and deaths. The majority of states in the USA, 32 in total, had significant increases in their suicide rate among adolescents and young adults aged 10–24 from 2000 to 2018 of between 30 and 60% (Curtin et al., 2021).

The U.S. Surgeon General’s Advisory (2021) on Protecting Youth Mental Health called attention to the COVID-19 pandemic's harm to the mental health of America's youth and families, as well as the mental health challenges that had accumulated before the pandemic began. Despite this increase in mental health challenges and rising rates of suicide, almost half of adolescents with mental health issues do not receive any mental health services indicating a growing gap in access to care (Whitney & Peterson, 2019). Disparities in access to care exist across geographic locations. They are exceptionally notable for children who are Black, indigenous, and people of color (BIPOC), children of undocumented immigrants, and those in low-income communities (NASP, 2021).

In response to the growing youth mental health crisis, schools have emerged as ideal and equitable venues for providing mental health interventions. Recent data from the National Survey on Drug Use and Health (NSDUH) show that youth are as likely to receive mental health care from schools as from outpatient mental health settings (Ali et al., 2019). Schools are also vital resources for racial and ethnic minorities and low-income youth with mental health needs and suicide risk. These groups are significantly more likely than white and higher-income youth to receive treatment only in educational settings (Ali et al., 2019).

With the grant support of SAMHSA Garrett Lee Smith and Project AWARE (Advancing Wellness and Resiliency in Education) grants, State Education Agencies in the USA have implemented mental health training and gatekeeper suicide prevention training for educators and school staff. While an important step, the body of research on gatekeeper training programs as a public health approach to suicide prevention shows that many adults do not feel comfortable talking with young people in crisis about their risk and that most suicidal adolescents do not feel comfortable talking to an adult about the problems they are experiencing (Torok et al., 2019; Wyman et al., 2008). Systematic reviews reveal that suicide prevention gatekeeper school-based education programs targeting adults in schools did not find an impact on suicidal behaviors in young people (Mann et al., 2021; Torok et al., 2019). Recent research supports the benefits of directly training adolescents in addition to training adults in school settings (Torok et al., 2019). Programs providing direct training to high school students prevent suicide attempts more often (Mann et al., 2021).

Teaching adolescents how to recognize and respond if a friend is considering suicide aligns with the body of research on help-seeking, which has found that many adolescents will first disclose distress and seek support from a peer related to mental health challenges and stress (Michelmore & Hindley, 2012). Increasing adolescent mental health literacy, or the knowledge and beliefs about mental disorders that aid their recognition, management, or prevention, is identified as a means of addressing stigma and improving access to treatment (Jorm et al., 1997). Both the World Health Organization (2019) and the U.S. Department of Education (2021) in guidance have called for increased focus in schools and communities on mental health literacy. Low levels of mental health literacy contribute to the misperception of mental health needs and reduced access to services (Miles et al., 2020), whereas gains in mental health literacy are associated with increased help-seeking intentions and potential treatment utilization (McCance-Katz & Lynch, 2019).

The challenge for schools in the USA has been finding a universal school-based intervention that improves adolescent mental health literacy and provides suicide and crisis prevention skills (Solerno, 2016). Universal school-based interventions engage the entire population of students in a setting. Many existing interventions have a targeted approach focused on supporting young people experiencing depression and suicide ideation (Busby et al., 2020). Other interventions focus on teaching students perceived as leaders in suicide prevention and helping skills (e.g., Sources of Strength) (Wyman et al., 2010). A universal approach to mental health education that enhances the existing health education in schools is optimal as it places value on all adolescents learning about mental health, the signs and symptoms of a mental health challenge, and how to help themselves or a friend if they are experiencing a mental health challenge or crisis.

## teen Mental Health First Aid

The teen Mental Health First Aid (tMHFA) program was developed in Australia in 2012 in recognition of critical adolescent mental health needs and with an understanding that young people prefer to go to their peers with problems. A Delphi consensus study provided essential information, and skills teens need to help a friend experiencing a mental health challenge (Ross et al., 2012). From there, guidelines for adolescents providing Mental Health First Aid to peers were developed into the tMHFA curriculum (Hart et al., 2012). tMHFA teaches young people in grades 10–12 how to identify, understand, and respond to signs of mental illness and substance use disorders among friends and peers. tMHFA accomplishes this goal by teaching a five-step action plan that helps teens have supportive conversations with their friends focused on seeking a trusted adult to take over as necessary. tMHFA was developed as a universal program for delivery to an entire grade level to ensure everyone has access to the same critical mental health information. The Australian curriculum (herein *A.U. tMHFA*) was designed for delivery by trained adults in schools or community sites over three interactive classroom sessions of 75 min each, and it includes slides, teaching notes, videos, role-plays, class activities, and a student manual (Hart et al., 2016).

A randomized cluster crossover trial comparing tMHFA to Physical First Aid among adolescents was then conducted in Australia and found that young people receiving tMHFA were significantly more likely to report helpful first aid intentions, increased mental health literacy, and less likely to endorse stigmatizing attitudes about mental illness than those who received physical first aid training (Hart et al., 2018). Hart and colleagues (2020) analyzed helping behaviors related to suicidal thoughts and behaviors. They found that tMHFA increased teens’ recognition of and intentions to assist a suicidal peer up to a year post-training.

## Current Study

Given the success and efficacy of tMHFA in Australia and the need for universal school-based programs in the USA to address adolescent suicide and depression, the National Council for Mental Wellbeing (formerly the National Council for Behavioral Health and hereinafter referred to as “National Council”) partnered with Lady Gaga’s Born This Way Foundation (BTWF) to adapt and implement tMHFA in the USA in 2018. This study was initiated to determine: how to retain the elements of the course that were evidence-based and effective while adapting the program for US students; what topics to add so US students have the essential information and skills teens needed to help a friend experiencing a mental health challenge or crisis; what changes to make to curriculum materials to ensure the style and delivery resonate with US students; and what tools to include so the program is implemented safely and with fidelity in diverse US schools.

This study used a contextual and cultural adaptation of the evidence-based A.U. program to best suit delivery in the context of US schools and youth organizations. In their paper on cultural adaptations of evidence-based interventions, Castro et al. (2010) define a cultural adaptation as "the systematic modification of an evidence-based treatment (or intervention protocol) to consider language, culture, and the context in such a way that it is compatible with the client's cultural patterns, meaning, and values.” This study, therefore, sought to examine how the curriculum and materials of tMHFA could be modified to be compatible with the language, help-seeking patterns, and mental health values of teens in the USA. This study engaged key stakeholder groups: youth, subject matter experts (SMEs), and Youth Mental Health First Aid (YMHFA) instructors to inform the process.

Given the global need for school-based mental health curricula, herein we report the adaptation process, including engaging participants, analyzing data, identifying key themes, translating the themes into actionable recommendations for course modification, and then making changes to the tMHFA program. By documenting this process, the authors hope to facilitate adaptation, uptake, and evaluation of the program in other cultures and contexts.

## Methods

### Study Design

This study employed a multimethod approach to adapt an evidence-based program from A.U. for use in the USA. The process involved recruiting and engaging diverse stakeholders in a feedback process, analyzing data, identifying key themes, synthesizing feedback from three stakeholder groups, and recommending adaptations for the tMHFA US curriculum.

### Participants

Study participants (*N* = 171) included youth, YMHFA instructors, and SMEs across the USA. The National Council emailed leaders from schools and organizations already implementing YMHFA to help recruit youth to participate in the study. Interested youth contacted the research team, who explained the study, and all youth submitted a parent/guardian consent form and verbally assented to participate in the research process. A total of 66 youth participated in the study. There were 20 subject matter experts (SMEs) invited to participate based on reputation in the behavioral health field as leading experts in the topic areas of interest (addiction, trauma, and school violence) through engagement with the National Council on other initiatives, relevant conferences presentations, and publications on the topic, or as recommended by a national professional association. The National Council emailed all YMHFA instructors (approximately 8500) and invited them to participate in a survey to inform the adaptation of the tMHFA curriculum. From the group, 87 YMHFA instructors completed the survey.

### Procedures

Five youth focus groups (two in-person in Washington, DC, and New York City and three virtual) with (*n* = 54 youth) from across the USA were facilitated by National Council and BTWF staff. Two focus groups were facilitated in person in Washington, DC, at a community-based mental health organization and one at a public high school in New York City. The other three were done virtually over Zoom on various dates and times to accommodate varying youth schedules. The focus groups lasted approximately 75 min and were followed by an anonymous survey via SurveyMonkey, eliciting demographic data and feedback about the focus group experience. Youth were guided through a slide deck with discussion questions during the focus group. The slide deck included sample language, scenarios, and images from the A.U. curriculum for the teens to react to, such as the action plan poster, which included comic book-style characters to depict each of the associated action steps. The primary topics covered in the focus groups included: how to make the scenarios, images, and language most relevant to youth in the USA. The team also inquired about what youth are currently learning in US school curriculum about mental health, suicide, substance use, trauma, bullying, and school violence; messages youth receive from American culture, media, and social media about mental health, mental illness, and help-seeking; the influence of social media on mental health, how youth would help a friend find a mental health professional and where youth in the USA go to get information about health and mental health; and ideal qualities an instructor needs to engage students best. Youth were not asked personal questions about their mental health. All youth focus group sessions were recorded and transcribed. Youth were provided a small honorarium for their time in the form of an Amazon gift card.

In addition, (*n* = 14) youth from a public charter school in Massachusetts were taught the A.U. tMHFA curriculum by an existing YMHFA instructor and school psychologist. After implementation, young people provided feedback in a focus group conducted by the research team over the video technology platform, Zoom, which asked young people to discuss what aspects of the course were most helpful, what content was confusing, what they would change so their US peers could relate and see themselves reflected in the scenarios, examples, images, and videos; and additional topics they would like to see taught in the course. These students also completed an anonymous survey via SurveyMonkey with demographic and closed and open-response questions about the curriculum’s look and feel.

Nineteen SMEs participated in the study over eight weeks, providing expert feedback on existing A.U. and potential new course content to further inform the adaptation of A.U. tMHFA for use in the USA. The SMEs met in two virtual sessions with the lead author. Before these sessions, SMEs reviewed all the A.U. tMHFA curriculum materials and provided detailed feedback on an electronic survey administered via SurveyMonkey. The survey asked participants to evaluate the A.U. curriculum using a rubric to score the strength of the scientific content, the effectiveness of the proposed delivery methods, and the relevance of scenarios, images, and videos to the lives of US youth. Next, SMEs identified what information was most critical for adolescents in the USA to know on the topic based on their understanding of youth development, the American education system, and cultural factors that may influence their understanding of the topic. Participants ranked the relevance of applying the A.U. tMHFA action plan to mental health and crisis situations. Finally, participants provided listed topic-related resources that could be shared with students, parents/caregivers, instructors, or school staff. Feedback from the surveys was compiled and presented to each panel for further discussion in a focus group, with conversations continuing until consensus statements were reached about what content was most relevant for young people in the USA to know and any cultural nuances that should be factored into the curriculum and by instructors when teaching young people about the impact of trauma, addiction, and school violence. Each SME was provided a small honorarium for their time.

Eighty-seven YMHFA instructors participated in the survey, administered through SurveyMonkey, which included demographic information such as MHFA role, state, gender, and race/ethnicity. Participants were asked what essential information and skills US youth need to help a friend experiencing a mental health challenge or crisis. They ranked 15 mental health-focused topics (e.g., stigma, self-care, specific disorders, suicide, trauma, bullying, etc.) based on their experience teaching the YMHFA course to adults working with young people in US schools. Participants were also asked open-ended questions related to what cultural considerations were essential to address in the US adaptation to ensure the program was relevant for diverse youth in the USA. Participants were asked to provide input on their experience with US school schedules, safety planning, the utility of possible implementation tools, and recommendations of US resources to provide in the training package for parents/caregivers, administrators, and school staff.

### Data Analysis

The qualitative data gathered during the focus groups with youth included notes taken by the facilitators and audio recordings from each session. The research team analyzed all data using NVivo qualitative data software. Transcripts, notes from the focus group, the debriefing session, and summary comments from the facilitators were all uploaded into NVivo. The team followed a six-step thematic analysis process (Braun & Clarke, 2006). (1) The lead author familiarized herself with all the data. (2) The lead author created a set of initial codes in a codebook within NVivo with a descriptor. Members of the research team confirmed the initial codes made sense, read through the transcripts and notes highlighting excerpts from the text, and applied the appropriate codes to them, identifying emerging codes for data not captured by the initial list and removing codes not used. NVivo software allowed the team to collate all the content. (3) The codes were sorted into themes and sub-themes. (4) The team then reviewed all the themes and summarized them related to answering the research questions about what content should be added, removed, or modified. (5) The team then created a thematic map of the data and focused on refining and defining each theme. At this stage, the team analyzed the consistency of themes across all six youth focus groups. This process allowed the team to assess if the themes from one group also emerged from other groups, ultimately reaching data saturation. (6) The research team developed a final report summarizing the key themes from the youth focus groups.

Data collected from the experts were collected and analyzed in two phases. First, the survey data participants completed giving their initial impression of the tMHFA curriculum and indicating whether they would endorse teaching US students the action plan steps to respond to specific situations involving their areas of expertise (substance use, traumatic experiences, or violence/ bullying). The results were analyzed using descriptive statistics within the SurveyMonkey platform, and reports were shared with each team of experts for discussion. The topics without 100% consensus from group members were added to the focus group agenda and discussed to get additional insight into the views of the participants who disagreed with the majority and to see if consensus could be reached. The focus group was recorded and transcribed, and notes were taken by the facilitators. The transcripts and notes from the focus group were analyzed using NVivo and followed the same six-step thematic analysis process (Braun & Clarke, 2006) used to analyze the youth focus group data. The only additional step taken in the process was sharing the final summary report with the group of experts. All participants confirmed that the findings accurately reflected the group's feedback.

Instructor survey data for closed-ended questions were analyzed using descriptive statistics within the SurveyMonkey platform. Frequency counts were run on each of the suggested content topics to add. Open-ended questions asking participants for additional content suggestions were analyzed using word count frequency. The team considered all suggested content topics with five or more responses. They included them in a final summary of recommendations for what to add to the curriculum, remove, and keep, but modify with additional descriptors of suggested modifications.

The research team summarized all the data into a final report that included the key themes from across each stakeholder group, noting similarities and differences related to what to add, remove, or modify. This report was sent to the National Council team, who made all decisions about addressing the curriculum changes and related training materials.

## Results

### Participants

This study engaged (*N* = 171) participants, including youth, MHFA instructors, and content area experts. Characteristics of each group of participants are described below in more detail.

Youth**:** A total of (*n* = 66) youth were engaged in the study. As indicated in Table 1, the youth participants were from fourteen states, with the most significant representation being from the eastern states (M.A., NY, DC). The 66 youth participants were all in the 10th through to 12th grade, with a roughly even split across each year level. There were slightly more females than males, but the group represented diverse geographic and racial/ ethnic backgrounds.

**Table 1:** Youth demographics (*n* = 66)

Description	*n*	%
*States represented*		
MA	14	21.21
NY	11	16.66
DC	8	12.12
TX	5	7.57
FL	5	7.57
VA	3	4.54
WA	3	4.54
OR	3	4.54
CA	3	4.54
IL	3	4.54
GA	2	3.03
WV	2	3.03
CO	2	3.03
MT	2	3.03
*Grade level*		
10th	24	36.36
11th	22	33.33
12th	20	30.3
*Gender*		
Female	38	57.57
Male	28	42.42
*Race/ethnicity*		
Non-Hispanic White or Euro-American	28	42.42
Black, Afro-Caribbean, or African-American	11	16.66
Latinx or Hispanic American	8	12.12
East Asian or Asian-American	5	7.57
South Asian or Indian American	3	4.54
Middle Eastern or Arab American	3	4.54
Native American or Alaskan Native	1	1.51
Not identified	7	10.6

### Subject Matter Experts

Subject matter experts (*n* = 19) participated in the study. As indicated in Table 2, the 19 S.M.E.s that participated represented a range of health and mental health professions, with the most being engaged in school and clinical psychology. The majority of participants (14) identified as non-Hispanic White or Euro-American, and there were more females than males, but there was representation from diverse geographic and racial/ ethnic backgrounds.

**Table 2:** Subject matter expert demographics (*n* = 19)

Description	n	%
*States represented*		
CA	3	15.78
DC	3	15.78
MD	2	10.52
NY	2	10.52
VA	2	10.52
MA	1	5.26
GA	1	5.26
RI	1	5.26
PA	1	5.26
FL	1	5.26
NE	1	5.26
CT	1	5.26
*Professional background*		
School psychologist	4	21.05
Clinical psychologist	4	21.05
Public policy researcher	2	10.52
Clinical social worker	2	10.52
Psychiatrist	2	10.52
School counselor	2	10.52
Pediatrician	1	5.26
Clinical counselor	1	5.26
School social worker	1	5.26
*Gender*		
Female	14	73.68
Male	5	26.31
*Race/Ethnicity*		
Non-Hispanic White or Euro-American	14	73.68
Black, Afro-Caribbean, or African-American	2	10.52
East Asian or Asian-American	2	10.52
Latinx or Hispanic American	1	5.26

### YMHFA Instructors

Stakeholders with extensive experience teaching existing MHFA curricula for adults in supporting youth in the USA, known as YMHFA instructors (*n* = 87) were included as participants. The group included National Trainers, i.e., contracted expert Youth MHFA facilitators that certify instructors to teach (*n* = 9) and YMHFA instructors (*n* = 78). Participating YMHFA instructors work in or with schools and youth-serving organizations from over 30 states. The states with the most representation included eight from New York (9.2%), seven from Texas (8%), six from Florida (6.9%), five from Colorado (5.7%), and five from Illinois (5.7%). Table 3 provides additional demographic information about these 87 participants. There were more females than males and representation from diverse racial/ ethnic backgrounds.

**Table 3:** Instructor demographics (*n* = 87)

Description	*n*	%
*Background*		
YMHFA instructor	78	22.22
National trainer	9	22.22
*Gender*		
Female	55	63.21
Male	32	36.78
*Race/ethnicity*		
Non-Hispanic White or Euro-American	42	48.27
Black, Afro-Caribbean, or African-American	16	18.39
Latinx or Hispanic American	11	14.1
East Asian or Asian-American	6	6.89
South Asian or Indian American	4	4.59
Native American or Alaskan Native	3	3.44
Not identified	5	6.25

### Process Outcomes

An overview of crucial feedback provided in the process by the participant groups and the corresponding adaptations made to tMHFA are provided in Table 4. In the table are summaries of the recommendations participants made by content area: addiction, school violence, trauma, social media, and self-care. Stakeholders also recommended that course materials better represent the diversity of youth in the USA, more instructional time be added for the new content to enable more interactive class exercises and discussion, and new implementation resources be developed to support use in US schools. Appendix A presents additional details about each participant group's recommendations.

**Table 4:** Key recommendations and adaptations made to the US tMHFA curriculum

Recommendation	Stakeholder group(s) who endorsed	Adaptation(s) made in response
Adapt content about addiction and substance misuse because = the US education system often is grounded solely in abstinence messaging	SMEs Youth YMHFA instructors	The content was added on substance use disorders, their prevalence, how to recognize the signs of substance misuse, and how to apply the tMHFA Action Plan (1) *Look for warning signs; *(2)* Ask how they are doing, *(3)* Listen up, *(4)* Help your friend connect to a trusted adult, *(5)* Your friendship is important*) to help a friend misusing substances and what to do in a substance use crisis
Add content about trauma and traumatic stress, given the prevalence of adverse childhood experiences in the USA	SMEs Youth YMHFA instructors	Content was added on what trauma is, examples of traumatic experiences, to explain how traumatic stress can impact mental health, and how to help a friend who experienced a traumatic event. Given the high percentage of US students who have experienced trauma, resources were added to help instructors use a trauma-sensitive facilitation approach
Add content about school violence to reflect the prevalence in the USA and youth fear of gun violence	SMSs Youth YMHFA instructors	Content was added on forms of school violence, how it can impact youth, and what to do if a peer is at risk of perpetuating violence as it relates to the tMHFA action plan and getting a trusted adult involved immediately, and calling 9-1-1
Adapt content about how social media and technology influence the mental health of US youth	SMEs Youth YMHFA instructors	Content was added on how social media and technology can impact youth mental health and how young people can recognize signs that a peer is struggling with their mental health via social media
Adapt content about US cultural factors that increase mental illness stigma	Youth YMHFA instructors	Additional content about stigma was included to explain how stigma can act as a barrier to youth getting help for themselves or a peer, as was a discussion of stigmatizing words related to mental illness and actions students can take to reduce stigma around mental illness in their school and community
Add content about self-care/ self-love given importance to US youth	Youth YMHFA instructors	Content was added about self-care/self-love to encourage students to take care of their mental health and manage stress. An activity was added where students created their self-love action plan
Enhance diverse representation of youth in the course to reflect US young people and ensure culturally responsive teaching of the program	SMEs Youth YMHFA instructors	Images and scenarios that depicted and represented diverse US youth of multiple races, cultures, genders, sexual identities, family backgrounds, and lived experiences of mental illness were added. This process included remaking A.U. tMHFA videos and adding new artwork and stories to feature diverse US youth. Content on culturally responsive teaching was also added to the instructor training
Adapt safety protocols to ensure US schools can safely implement the program	SMEs Youth YMHFA instructors	An Exit Ticket process was added to give instructors a mechanism to assess students’ well-being discretely after each session
Create USA-specific implementation guidance	YMHFA instructors/	An Implementation Toolkit was designed to provide US schools and organizations with best practices and strategies for safely implementing the tMHFA program from start to finish with fidelity
Add educational resources for parents/caregivers who do not undertake MHFA training	Youth YMHFA instructors/	A video was developed for parents/caregivers who may be unavailable to take a YMHFA offered at the site about the tMHFA program. It included information on how to have supportive conversations with their child about mental health, resources, and what to do in a crisis
Alter time and flexibility of format to ensure adoption in the US school system	Youth YMHFA instructors	Forty-five minutes were added to the course to create time for covering the added US content. Two formats for teaching the course were developed (6 sessions of 45 min or three sessions of 90 min) to give sites options for aligning the course to their schedules

### Addiction and Substance Misuse

All participant groups agreed that more content about addiction and substance misuse was needed to increase the relevancy of the A.U. tMHFA curriculum for the USA. This recommendation was grounded in knowledge of the opioid epidemic in the USA and patterns of substance use among youth in the USA; for example, 38% of 12th graders and 30% of 10th graders reported an illicit drug in the USA in 2019 (NIDA, 2021). Both youth and YMHFA instructors noted that while some education about addiction and substance use was provided in US schools, most was not evidence-based, emphasized abstinence only, relied heavily on scare tactics, and left the youth with questions about what to do in a crisis. Therefore, the US tMHFA curriculum was modified to expand on the topic of addiction, teaching how substance use can impact brain development and how common substance misuse is among teens. The US course retained modeling the recovery position from the A.U. tMHFA crisis section but added content about responding to an opioid overdose (i.e., introducing Naloxone and explaining where to get more education about using it).

### Trauma and Traumatic Stress

All participant groups agreed that the US tMHFA curriculum should include information on trauma, its impact on mental health, and what to do if a peer experiences a traumatic event. The US tMHFA curriculum was thus altered to include examples of traumatic experiences, teach how individual responses to traumatic stress can vary by person, and explain how the impact of trauma can be modified through the receipt of help and support. In the crisis section, specific information was added about how to help a friend who has experienced a traumatic event such as violence. Resources and information were also added to help instructors teach tMHFA in a trauma-sensitive way by establishing routines, communicating expectations, providing opportunities to take breaks, and validating participants’ lived experiences related to the material.

### School Violence

All participant groups agreed that the tMHFA curriculum needed to include a discussion on school violence, other types of violence, and how to apply tMHFA action steps (Table 4) if a student was worried about a peer being a victim of violence or committing violence against someone else. The tMHFA A.U. course addresses bullying but not school gun violence. This recommendation for including school violence content was supported by national US data, indicating that young people experience violence at high rates in and out of school, which is a significant source of stress. In a national survey by the American Psychological Association (2018), three out of four teens in the USA said that worrying about gun violence, mass shootings, and school shootings was a significant source of stress.

Notably, marginalized groups in the US experience violence at disproportionately higher rates. For example, in US schools, 43% of transgender youth have been bullied on school property, compared to 18% of cisgender youth; 29% of gay or lesbian youth and 31% of bisexual youth have been bullied on school property, compared to 17% of straight youth (Underwood et al., 2020). Black youth in the USA are at higher risk for the most physically harmful forms of violence, such as homicides, fights with injuries, and aggravated assaults, than white youth (Sheats et al., 2018).

Therefore, adaptations were made to the US curriculum to teach students about multiple forms of violence, including school gun violence, and how experiencing or witnessing violence can be traumatic. Instructor training was altered to explain how to validate concerns that students may have about school gun violence, often due to media coverage, while showing the rarity of these incidents and explaining what the school or organization is doing to keep young people safe. Changes were also made to teach students to use the tMHFA Action Plan to seek help from an adult immediately if concerned that a peer may be at risk of hurting themselves or someone else and to dispel the myth that all people with mental illness are violent.

### Social Media and Technology

All participant groups recommended adding content about social media and technology to increase the relevancy of the tMHFA A.U. curriculum to US youth, 95% of whom have access to a smartphone, and 45% say they are online "almost constantly" (Pew Research Center, 2018). YMHFA instructors noted the importance of including research on the harms of social media on youth mental health. Youth also suggested in focus group discussions that their relationship with social media was complex, with it sometimes being a source of connection and other times causing feelings of insecurity. The US tMHFA curriculum was adapted to prepare instructors to facilitate conversations about how social media impacts mental health. Materials were also added that provided young people with tips for noticing and identifying their emotions while using social media and technology and information on how to be intentional in their use. Information was also added on how to recognize signs on social media that a friend may be experiencing a mental health crisis, as was an opportunity to practice applying the tMHFA Action Plan (Table 4) to help a friend via text message.

### Mental Illness Stigma

Youth and YMHFA instructors recommended adding information on mental illness stigma to the curriculum. Instructors shared how frequently stigma: (a) arises as a topic of discussion during YMHFA courses and (b) is described by youth as the primary barrier to seeking help. The youth noted the role of cultural and family beliefs in understanding variations in stigma experiences among peers. Therefore, the US tMHFA curriculum was altered to introduce and define mental illness stigma, to discuss factors that impact stigma, and to allow students to understand and address it as a barrier to getting help for themselves or a peer. An activity was also added where stigmatizing words related to mental illness are discussed and reframed using more positive language, followed by a discussion on how they can reduce stigma around mental illness in their school and community informed by available resources.

### Self-love

Adding content about self-love or self-care was recommended by youth in the focus groups, who shared that this was not a topic they had learned about in any current high school course but wished they had. The youth noted that the tMHFA course focused heavily on how to help a friend and, while necessary, felt their peers would want more information on how to take care of themselves and improve their well-being. The National Council included this content based on growing research showing that adolescents benefit from many self-care practices studied in adults, including sticking to a schedule, eating well, staying physically active, getting quality sleep, staying hydrated, and spending time outside (National Academies of Sciences, 2019). The adapted US tMHFA curriculum was changed to introduce young people to the concept of self-love and its connection to mental health and explore strategies they can use to promote their physical, social, and emotional health. An activity was added where students make a self-love action plan that they are encouraged to refer to during the course if they feel stressed or overwhelmed. The US tMHFA curriculum also was adapted to acknowledge how helping a friend experiencing a mental health challenge can be difficult, encouraging young people to practice their self-love strategies to avoid taking on too much.

### Enhance Diverse Representation of Youth in the Course

All stakeholder groups recommended incorporating diverse youth voices into the US tMHFA curriculum and ensuring the course was taught culturally responsive. The National Council conducted a youth artwork campaign and received submissions from diverse youth across the USA on mental health for this purpose. The National Council also worked with a video production company to remake all three of the tMHFA curriculum videos to feature US youth from diverse backgrounds sharing their stories about recovering from mental health challenges. In addition, National Council added images and scenarios that reflected youth of different races, cultures, genders, sexual identities, abilities, locations, family backgrounds, and lived experiences of mental illness.

The National Council also addressed the instructor preparation to help instructors teach in a culturally responsive way. This process includes content about culture and how it can impact young people and their families understanding of mental health, mental illness, stigma, help-seeking, and treatment options. Instructor trainees engage in a facilitated discussion on how they can affirm the various identities of young people and create a safe space for all students while teaching the tMHFA course.

### Adaptation of Safety Protocols

Just as Mental Health First Aid Australia—the organization that delivers A.U. tMHFA to schools in Australia—has safety protocols for instructors and schools (Hart et al., 2016, 2018), the National Council encouraged schools to develop a student safety protocol. The National Council created a sample protocol for schools to use as a template if they did not have one already in place that included steps for reaching out to the teen and their parents/caregivers and referring them to a professional mental health clinician when necessary.

All participant groups noted the sensitive nature of topics covered in the tMHFA curriculum, e.g., suicide, and encouraged the National Council to enhance safety tools to ensure the safety of all students. In response, the National Council created a US tMHFA Exit Ticket process, a discreet way for schools and organizations to check on students' well-being after each training session. The Exit Ticket was designed to be distributed by instructors at the end of each session. Teens share their feelings, ask questions, and indicate if they want to be checked on by an adult. The Exit Tickets allow young people to contact the instructor for help. Instructors must read each Exit Ticket before teens leave campus for the day, and schools must have a protocol in place to support teens that indicate they want to be seen by a counselor or mental health professional.

### Creation of a US Implementation Toolkit

The A.U. tMHFA curriculum provides implementation guidance for instructors and schools to use the program in their local context (Hart et al., 2016, 2018). YMHFA instructors recommended modifying the A.U. guidance with additional resources for helping schools and organizations integrate the US tMHFA curriculum into their overall mental health promotion and crisis response plan. This guidance was recommended given the diverse nature of schools and organizations across the USA. The National Council created an Implementation Toolkit, a booklet designed to provide sites with best practices and strategies for implementing the program at a site from start to finish. It includes a checklist and sample timeline for getting buy-in from school or organizational leaders through presentations and informational letters, preparing other educators/staff at the site, informing parents/caregivers about the training, and getting students ready. It also includes teaching tips for instructors. Examples and stories from sites on best practices, common challenges, and how problems were overcome are interspersed throughout the toolkit. Information and resources for administrators and parents/caregivers about how to speak to their teens about mental health are incorporated, as are strategies for sustaining the program.

### Time and Schedule Flexibility

Based on participant feedback, The National Council created an option that better aligned with US school schedules. It allows them to teach the course in 3 sessions of 90 min each or six sessions of 45 min each to provide more flexibility and align with schools' block schedules. The course agenda was reworked to accommodate new content and more discussion and activities opportunities. Table 5 shows the comparison of the A.U. and US tMHFA (three-session model) curriculum by session, noting US adaptations.

**Table 5:** Comparison of A.U. and US tMHFA curriculum outlines*

A.U. tMHFA curriculum outline (three sessions of 75 min)	US tMHFA curriculum outline (three sessions of 90 min)
**Session 1: Introduction to tMHFA** Introduction to tMHFA The spectrum of health and mental health Relationship between thoughts, feelings, and behavior What are mental health challenges? The impact of mental health challenges on teens Professionals who can help Other people and supports that can help	**Session 1: What are mental health challenges?** Introduction to tMHFA The spectrum of health and mental health Relationship between thoughts, feelings, and behaviors What are mental health challenges? *What factors impact mental health challenges? *Influence of social media on mental health *Influence of trauma on mental health **Part 2: What is appropriate help?** The impact of mental health challenges on teens *The role of stigma and how to reduce it Professionals who can help Other people and supports that can help *Self-love
**Session 2: Mental health crisis** What is a crisis? Introducing the tMHFA action plan Look for warning signs Ask how they are Listen up Help your friend connect to an adult Your friendship is important Using the tMHFA action plan in a crisis involving suicidal thoughts/behaviors Other crisis: Self-injury Bullying and abuse Substance misuse Recovery position	**Session 2 Helping a friend in crisis** What is a crisis? Introducing the tMHFA action plan Look for warning signs Ask how they are Listen Up Help your friend connect to an adult Your friendship is important Using the tMHFA action plan crisis involving suicidal thoughts/behaviors **Part 2: Helping a friend in crisis** Helping a friend who is in another type of crisis: *Panic attack Self-injury *Traumatic event Bullying *Violence Using the tMHFA Action Plan in an above crisis situation
**Session 3: Developing mental health problems** How can you tell if someone is developing a mental health problem? Using the tMHFA Action Plan to help a friend experiencing a mental health problem Review of local and national mental health resources Recap of course	**Session 3: Helping a friend in a substance use crisis and helping a friend with a mental health challenge** What is a substance use crisis? ***Opioid overdose and Naloxone*** Using the tMHFA Action Plan to help in an overdose crisis Recovery Position How can you tell if someone is developing a mental health challenge? Using the tMHFA Action Plan to help a friend developing a mental health challenge **Part 2: Recovery and resilience** *Recovery and resilience Review of local and national mental health resources Recap of course

*Notes new topics added

### Resources for Parents/Caregivers and School Staff

To ensure that adults have the knowledge and skills to support students who seek help for their mental health, the implementation protocol for A.U. tMHFA requires that 10% of school staff are trained in the Australian 14-h Youth MHFA program and that YMHFA is offered to parents of students who will receive the tMHFA program. The US tMHFA program incorporated the exact requirement that 10% of school staff are trained in US YMHFA. However, the requirement that all parents/caregivers were trained in YMHFA was modified fr US tMHFA based on feedback from YMHFA instructors, who felt taking an all-day YMHFA course would not be feasible for many parents/caregivers in the USA. Instead, it was recommended that parents/caregivers be given credible information and resources on mental health and guidance on having a conversation about mental health with their child in an on-demand format. The National Council, therefore, created a brief video for parents/caregivers covering the basics about mental health, mental health disorders, what their child will learn in the tMHFA curriculum, tips for having a conversation about mental health, and signs their child may be experiencing a mental health challenge or contemplating suicide and where to get help. Information sessions were also designed to be held at schools for parents/caregivers and staff.

## Discussion

This study sought to garner feedback from multiple stakeholders (youth, SMEs, and YMHFA instructors). The tMHFA A.U. curriculum was adapted by adding and modifying content to meet the educational needs of American secondary students while retaining core components of the original evidence-based program. In addition, the study sought to identify any culturally based language/terms, examples, or resources that were only applicable to A.U. youth and replace them with scenarios, examples, images, and videos to ensure the American youth could see themselves reflected in the curriculum. The areas participants recommended be modified—substance use; school violence, trauma, and traumatic stress; social media and technology; mental health stigma; and resources for parents/caregivers and schools—were also each supported by data and literature indicating the importance of these domains for adolescent development in the USA.

The participants’ recommendation to enhance the content addressing substance use and overdose situations was justified, given data suggesting that in 2019, there were 4777 reported drug overdoses among youth in the USA (NIDA, 2021), and of those, more than three out of five deaths involved at least one potential opportunity to link people to care before or to implement life-saving actions before or during the overdose occurrence (O’Donnell et al., 2020). Statements made by youth and YMHFA instructors about the lack of evidence-based messaging on substance use are supported by research showing that while 80 percent of American youth reported participation in school-based prevention in 2005, only 20 percent were exposed to evidence-based prevention programs (Miller & Hendrie, 2008). The burden of substance use and overdose within communities of young people combined with low overall exposure to evidence-based substance use programs in school firmly demonstrates the need for explicitly providing psychoeducation on substance use, specific skills (e.g., the recovery position), and information to connect youth to community resources (e.g., Naloxone), within the tMHFA US curriculum.

The participants’ recommendation to add new content addressing violence, a topic unaddressed in the A.U. tMHFA curriculum, makes sense when contextualizing how much more frequently young people hear about and are exposed to violence or concerns of violence. The USA has had 57 times as many school shootings as all other major industrialized nations combined (Grabow & Rose, 2018). From 2019 to 2020, there were 75 school shootings with casualties across the USA (Irwin et al., 2021). Youth in the USA also commonly report other forms of violence and safety issues impacting their lives. Nearly nine percent of high school students did not go to school at least once during the past month because they felt unsafe on or on their way to or from school (Underwood et al., 2020). One in five American high school students reports being bullied at school each year, and one in six reports cyberbullying each year (Underwood et al.,^ 2020^).

Recommendations to add content on other trauma were supported by data indicating that one in four youth in the USA will experience at least one traumatic event by the age of 17, according to the Child and Adolescent Health Measurement Initiative (2016–2017). Notably, experiencing traumatic stress is a risk factor for nearly all mental health disorders without appropriate help and support (SAMHSA’s National Child Traumatic Stress Initiative (NCSTI), 2017). Further, youth of color and socioeconomically vulnerable youth in the USA are more likely to experience ACES. Nationally, 61 percent of Black non-Hispanic youth and 51 percent of Hispanic youth have experienced at least one A.C.E., compared with 40 percent of white non-Hispanic youth and only 23 percent of Asian non-Hispanic youth (Sacks & Murphey, 2018).

The recommended content added to help students learn about social media's impact on mental health is aligned with research that has found social media use is linked to mental health challenges in youth, such as depression, anxiety, and psychological distress (Keles et al., 2020). However, 2018 survey data demonstrated that teens are more likely to indicate that social media makes them feel included rather than excluded (71% vs. 25%), confident rather than insecure (69% vs. 26%), authentic rather than fake (64% vs. 33%), and outgoing rather than reserved (61% vs. 34%) (Pew Research Center, 2018). tMHFA can serve as a vehicle for the provision of information to help students navigate a complex relationship between social media use and mental health, in addition to helping them extend the skills they learn on assisting a peer to online interactions. Given that there was no current information on the impact of social media on teen mental health in the original program, the tMHFA A.U. program authors consider this adaptation one of the most important innovations of the adaptation process. They will look to incorporate content on this topic in future curriculum updates.

The addition of content and active discussion on mental health stigma is supported by recent findings of a systematic review of qualitative and quantitative studies that identified perceived stigma and embarrassment as the second most common reason adolescents report not seeking professional help or treatment for mental health problems (Radez et al., 2021). Further research shows that race, ethnicity, and gender identities can affect how US youth perceive mental illness in themselves and others. DuPont-Reyes et al. (2020) found lower levels of mental illness knowledge and more stigmatizing attitudes among boys and members of racial and ethnic minority groups. They cautioned that differences in early life, prior to the onset of the most common and significant mental illness, can contribute to the disparities in mental health service utilization and recovery by people of color.

The inclusion of enhanced resources for parents or caregivers of students in tMHFA USA aligned with the original A.U. approach and a growing body of research showing that the most important thing a child needs to be resilient is a stable and committed relationship with a supportive adult (National Scientific Council on the Developing Child, 2015). It also aligns with early work by the developer of MHFA on the importance of offering a range of mental health literacy interventions at the community level (e.g., wellness campaigns, embedding services within educational programs, and providing information on Web sites) to empower families and community members to act for better mental health (Jorm, 2012). Mental health literacy training programs, such as YMHFA, can empower families with skills to recognize and assist their children experiencing mental health challenges and better prepare them to seek help.

There are some significant limitations to our approach. While a substantial group of stakeholders representing diverse geographic areas, experience levels, and familiarity with MHFA was engaged, some critical populations (e.g., non-English speakers or students for whom English is not their first language) were not included. Future versions of the tMHFA program should be translated into Spanish and other critical languages to reach more young people in the USA. Importantly, this will likely require additional cultural and contextual adaptation beyond language.

Specifically, there were limitations in the diversity of youth recruited. While the authors captured data about grade level, race/ethnicity, gender, and state, there were vital demographic data that could have further helped assess the diversity of the youth who participated, including sexual orientation, socioeconomic status, and geographic diversity (i.e., rural, urban, or suburban). In addition, young people who participated were not asked specially asked whether they were experiencing or had experienced a mental health challenge or had supported a friend or loved one with a mental health challenge. Gathering these data in future and ensuring a variety of viewpoints and experiences are represented in the sample is essential to ensuring the adaptation process captures all relevant issues.

There were also limitations in the diversity of the adult experts and YMHFA instructors recruited. The majority racially identified as white leaving a considerable gap in the perspective of participants of color. Another limitation was the professional experience of the experts. Many had expertise in youth mental health and had worked with and in schools during their early career, yet at the time of the study, more were employed by research and higher education settings, meaning they could not give a complete perspective on how this program might be received in school settings.

This adaptation process also occurred before COVID-19; thus, the feedback gathered does not reflect the added stressors and collective trauma young people in the USA have experienced during the pandemic (Samji et al., 2022). In addition, the adaptation process centered around delivering the course in person and did not seek out information on how to best deliver the course as a blended or online experience, which is relevant given the shifts in the range of ways schools offer. Students expect to be able to access instruction.

Despite these limitations, the process used to create the tMHFA US curriculum has essential strengths. Building upon lessons learned from a contextual and cultural adaptation of evidence-based interventions (Castro et al., 2010), the process incorporated an emphasis on using systematic and detailed feedback from program participants to guide adaptation. The process was also informed by studies in which other MHFA curricula have been adapted through multistage processes (Doyle et al., 2014) using methods such as Delphi consensus (Li et al., 2021), focus groups with stakeholders, and engaging expert reference groups (Wang et al., 2022). Using a multimethod approach allowed the research team to get a complete picture of the participants' perspectives.

By drawing on all these lessons, the authors created a process for generating and distilling recommendations into content changes or additions that were practical and timely. The adaptation process, which resulted in a complete curriculum ready for pilot testing with students, instructors, school staff, and parents/caregivers, took less than one year. Further, the close working relationship between the A.U. tMHFA research team and the US tMHFA team enabled insight into original design decisions and particulars of the evidence base to ensure the US program retained the core components (e.g., the action plan) that make tMHFA effective.

There are several important implications for future research on tMHFA in the USA. First, the efficacy of the adaptation process will be measured in future research that assesses the course’s impact on young people’s mental health literacy and reported ability to help peers struggling with a mental health challenge or crisis in the USA. Comparisons will be possible to see whether US students achieved similar mental health literacy outcomes as did students in Australia (Hart et al., 2018), which will help verify the rigor of the adaptation process. In addition, there is now a version of tMHFA in Australia for students in grades 7–9, allowing young people to get exposed to the curriculum twice throughout their education to bolster the effect. Given growing concerns about poor mental health outcomes and increased suicide ideation for younger students in the USA (Curtin et al., 2021), this may be important for tMHFA USA to include, as well. However, adaptations needed for this age group may differ from those for older students and will need further exploration. tMHFA was designed to be scalable and sustained within schools and organizations, but given variations in the school context, the cultural understanding of mental health, and the types of problems youth face in different settings, adaptations are necessary to ensure program success. Ultimately, this study’s findings demonstrate the adaptations needed to facilitate the implementation and maintenance of program effectiveness as tMHFA is introduced to new populations. In addition, the outlined process can be replicated for this purpose as the program continues to expand in the USA and other countries. This study indicates that more attention should be given to the stakeholder recruitment process and ensuring the participants selected represent the diversity of the country's young people and that SMEs engaged come from a range of professional backgrounds with expertise in adolescent mental health and development.

## Appendix A: Recommendations and Feedback from Stakeholders

### Initial tMHFA Workgroup

Three primary recommendations emerged from the workgroup review activities of the initial workgroup. First, there was consensus that the course should address substance use and addiction more prominently. tMHFA A.U. included limited information about addiction, substance misuse, and responding to a crisis involving substance misuse or overdoes since that information is commonly covered in the health education curriculum. The workgroup recommended that since the information about substance misuse in school varied widely across the USA, the most critical knowledge and information for adolescents to know about substance use should be added. Secondly, the workgroup flagged the importance of understanding and addressing trauma as a risk factor for mental health challenges in young people in the USA within the tMHFA curriculum. Specifically, the workgroup recommended that the topic of trauma, traumatic experiences, and their impact on mental health should be further reviewed by experts in the field to determine what knowledge and information were most critical for adolescents to know if a traumatic event occurred or if they or a friend experienced one or more in the past.

Relatedly, the third key recommendation from the working group was that the course needed to address better the impact of violence in the lives of US youth. The workgroup noted that a significant difference between US and A.U. high school young people experience is related to community and school violence, particularly the threat of gun violence and mass shootings. The A.U. tMHFA curriculum addressed the impact of bullying, but the workgroup recommended that this should be expanded in the USA. The workgroup noted the importance of avoiding perpetuating stereotypes about people with mental illness bring prone to violence while acknowledging the heightened stress and anxiety that adolescents report feeling about the threat of violence: three out of four teens in the USA said that worrying about gun violence—mass shootings and school shootings—is a significant source of stress (A.P.A., 2018). Nearly 7% of high school young people did not go to school at least once during the past month because they felt unsafe either at school or on their way to or from school (Kahn et al., 2018).

### High School Student Focus Group Participants

Overall, adolescent focus group participants generally seemed enthusiastic about training that would teach them skills to identify a mental health or substance use problem in a peer and that would empower them to get that friend's help. Participants indicated that while schools do tend to provide some information about mental health problems and substance use, it is not continuous or in-depth. Young people indicated variation in how or to what extent the focus group topics were addressed in their current school curriculum or environment; substance misuse was covered in most but to varying degrees, bullying was often said to be discussed in assemblies or via posters, and suicide/self-injury was sometimes covered in health class. Young people provided additional feedback about the culture, resources, and complexity of the topic of mental health, such as the impact of trauma and how it can impact young people differently. They recognized the importance of addressing the impact of trauma on young people, risk factors, recovery/resilience, and school violence as essential to include. In addition, there was general agreement that information on substance misuse, including resources to help their peers struggling with the use of substances other than alcohol and marijuana, how to respond to an opioid overdose, and strategies beyond just saying no, was needed. Several areas of more specific feedback were also given, as detailed below.

Materials: Most young people felt that the comic scenario was helpful and realistic, but some felt that the scenario moved too fast and that a few steps should be added. Feedback on the action plan’s graphics included a feeling that there was forced diversity and characters were stereotypical, but most felt indifferent. Focus group participants noted the need to include diverse representation in all the course materials, from the manual's images to the stories told in the curriculum videos.

Instructors: Young people mentioned that instructors should be open and honest, good listeners, make a personal connections, and ask for additional input.

Social Media: Young people flagged how much their lives centered around social media and wondered whether the course could talk about the benefits of social media and the negative ways it can impact mental health.

Self-Care: Participants noted that they wanted more information about self-care and felt the course focused too much on helping a peer that they wanted information about taking care of themselves.

### Topical Expert Panels

Nineteen individuals were engaged in this process, including six school violence experts, six trauma experts, and seven substance use experts. All experts, except for one substance US expert, expressed via the survey a feeling that the tMHFA curriculum would be helpful for young American people. On average, experts gave the course 82 out of 100 for usefulness. In addition, 88% of experts agreed that the tMHFA guidelines developed in Australia that served as the curriculum's basis were relevant for young American people providing mental health first aid to a peer. All experts also agreed with the steps of the tMHFA Action Plan (Look for Warning Signs, Ask how they are, Listen up, Help them connect with an adult, Your Friendship is Important) and that the utility of the curriculum would be improved by incorporating more information on the given topic area of focus for the panel (substance use, trauma, and violence). Their suggestions to make the curriculum more valuable and relevant for teenagers in the USA included adding more diversity and lived experiences into the examples; discussing triggers and the role of social media; including examples of trauma, substance use disorder, and a range of school violence scenarios; and adding additional pre and post resources.

Trauma Panel: The six trauma panelists suggested ways to integrate more trauma-related and trauma-sensitive content, including acknowledging that many teens have likely experienced traumatic events and introducing the definition of trauma, examples of trauma, and strategies for promoting resilience. The trauma expert's consensus statements included: the definition of trauma, an example of traumatic events, signs and symptoms of peers in psychological distress and impacted by trauma, connecting the peer to a supportive adult, and defining resilience. Experts emphasized the importance of proceeding with a trauma-sensitive approach.

School Violence Panel: The six school violence prevention experts suggested several pieces of content and methods for integrating content on preventing school violence and awareness of peers who are not only at risk of harming themselves but may also be at risk of harm to others. First, they described a need to avoid using a fear-driven approach (e.g., debunking the myth that school shootings are frequent and increasing) and ensuring violence prevention discussions are broad enough to include bullying, abuse, sexual harassment, dating violence, not just school shootings. The emphasis was suggested on healthy behavior and relationships and where to go for help. The panel recommended building out scenarios and group discussions involving how to respond to a peer threatening to harm others, together with information on a student considering suicide, given that the warning signs can often be the same. Specifically, they felt the question "Have you ever thought about killing yourself?" could be followed by "are you considering hurting someone else?" The school violence panel also felt it was important to clarify that young people should report concerns to administrators and not personally investigate issues. It emphasizes that decision making should be based on compassion and understanding, not fear.

Lastly, the panel recommended addressing stereotypes about people with mental illness being violent directly, explaining that someone with a mental illness does not carry out most acts of violence, perhaps even with a myth versus fact exercise or handout. Ultimately, the school violence prevention expert panel consensus statements included: understanding violence, signs a friend is in distress (at risk for harming self or others), reaching out to a friend and asking how they are, reporting any concerns to an adult at school, and build connections and practice empathy.

Substance Use and Addiction Panel: The substance use and addiction experts suggested the following ways to integrate additional content on substance use and addiction: building out substance use information from the start of the curriculum; explaining the relationship between substance use and mental health, including discussion of co-occurring disorders; acknowledging that substances such as alcohol, opioids, and cannabis and misused prescription medications can be dangerous and have implications for mental health; and clarifying what symptoms of overdose are and what substances may cause an overdose. In addition, the experts agreed on the importance of including emergency procedures for substance use crises, including calling 911; listing protocols for helping someone who may have overdosed; reviewing the use of NARCAN; and determining and sharing what local resources are available to teens in their area. Lastly, if possible, the experts felt that the videos should include the story of a young person with a substance use disorder or someone who talks about using substances as a coping mechanism. The substance use and addiction expert panel’s consensus statements included: what are substance use disorders and how prevalent are they, substance use has consequences and can lead to severe problems, recognize the signs of a substance use problem, using substances may be an indicator of other mental health problems, recognize the signs a friend may be in crisis and what to do and how to get help, and teens can get help with addiction.

### Recommendations from Instructors and National Trainers

Three key recommendations emerged from the feedback from the instructors and National Trainers related to stigma, student safety, and implementation considerations. **Firstly, the survey participants** noted how frequently stigma comes to the USA during the course and what a significant barrier stigma still is in many cultures and communities. Participants recommended unpacking the concept of stigma surrounding mental illness to allow young people to understand and address it as a barrier to getting help for themselves or a peer. Secondly, they stressed the need for adequate preparation of tMHFA instructors and schools to monitor the psychological well-being of young people during and after the course. Survey participants expressed concern that the sensitive course content could cause young people to experience distress without having a way to ask for or reach out for support. Lastly, survey participants noted that school schedules varied considerably across the country, and for the tMHFA US course to be successful in being implemented widely, there would need to be some flexibility in the way the course was structured.

## Recommendations from J.H.U. Research Team

In addition, they were echoing many of the points raised by the last panels, the J.H.U. The team focused on providing guidelines to ensure this could be implemented within high schools and how to bring together the final package of materials in a way that could reach the US audience (e.g., ensuring a match to US youth language and experiences in content, parent content). The recommendations for implementation included providing structured suggestions such as implementation during health classes; allowing for sessions to be either 60 or 90 min; identifying possible second staff members to serve as “welfare staff” (i.e., to check in with young people during or after the sessions) during the training and creating plans for how to respond when young people showed signs of distress; identifying points of contact at each site to facilitate the communication with the community. Regarding content, the J.H.U. provided suggestions on language (e.g., specific words that would not be as understandable to US youth, attuning to reading levels of the content, and updating information like suicide statistics and resources) and visual content (e.g., art, cartoons) across materials. They suggested building in the opportunity for young people to rehearse, asking a friend if they are suicidal, and talking to youth about mandated reporting and confidentiality. The team also suggested building a way to assess for possible iatrogenic impacts of the tMHFA program.

## Appendix B (See Table 6)

**Table 6:** Comparison of tMHFA implementation guidance between Australia and the USA

	Australia	USA
tMHFA instructor requirements	tMHFA instructors are required first to be qualified as a YMHFA instructors, meaning they have been through 5.5 days of training and are qualified to present the 14-h youth program to adults living and working with adolescents	Instructor candidates who want to be trained to deliver tMHFA to students must also be trained in the Youth Mental Health First Aid course before attending the tMHFA instructor certification course. Instructor candidates do not need to be certified to teach Youth Mental Health First Aid to be eligible to teach tMHFA
Training staff on-site in YMHFA	Suppose tMHFA instructors are not qualified YMHFA instructors. In that case, they must partner with an existing YMHFA instructor to ensure they can deliver the whole suite of MHFA training to the school communities they are providing tMHFA to. tMHFA instructor training for these individuals is longer and involves more information on student engagement and safety than those who have already undertaken YMHFA training	Before teaching tMHFA to students, the training location must have 10% of adult staff trained in Youth Mental Health First Aid
Parent/Caregiver Information	TMHFA instructors give information briefings and newsletters to parents and teachers who have yet to undertake YMHFA training. Hence, they understand the content of the tMHFA program for adolescents and how to have supportive conversations about student mental health	Sites are encouraged to provide Youth Mental Health First Aid training to parents and caregivers before the tMHFA course begins or concurrently with the tMHFA course
Student training requirements	The program must be taught to students in grades 10, 11, or 12. tMHFA for 6–8 may be taught to lower grades The program must be taught to an entire grade level (not individual classes) in a school or to an entire group of students at a youth-serving organization or youth program	The program must be taught to students in grades 10, 11, or 12. At this time, grade 9 is not permitted The program must be taught to an entire grade level (not individual classes) in a school or to an entire group of students at a youth-serving organization or youth program It is recommended that sites have one teen Mental Health First Aid instructor for every 100–150 students. (Instructors will not be teaching students in extensive, assembly-style sessions. This recommendation is meant to help sites gauge how many instructors should be trained in the curriculum.)
Student safety		Sites offering the training must meet criteria to ensure student safety by having a mental health professional on-site. At the same time, the course is being taught as a protocol to respond to distressed students Sites must also commit to using tMHFA Exit Tickets to give students a discreet way of asking for help for themselves or a friend after every course session Sites must establish a protocol to have instructors and a site staff person read every tMHFA Exit Ticket and contact every student who requests help
